# Use of nanotechnology for infectious disease diagnostics: application in drug resistant tuberculosis

**DOI:** 10.1186/s12879-019-4259-x

**Published:** 2019-07-12

**Authors:** Roshanthi Eranga Karunaratne, Lahiru A. Wijenayaka, Sandya Sulochana Wijesundera, K. M. Nalin De Silva, Chamila Priyangani Adikaram, Jennifer Perera

**Affiliations:** 1 Department of Microbiology, Faculty of Medicine, University of Colombo, box 271, Kynsey Road, Colombo, PO 08 Sri Lanka; 2 Sri Lanka Institute of Nanotechnology (SLINTEC), Mahenwatte, Pitipana, Homagama, Sri Lanka; 3 Department of Chemistry, Faculty of Natural Sciences, The Open University of Sri Lanka, Nawala, Sri Lanka; 4 Department of Molecular Biology and Biochemistry, Faculty of Medicine, University of Colombo, Colombo, 08 Sri Lanka; 5 Department of Chemistry, University of Colombo, Colombo, 03 Sri Lanka; 6 Central Public Health Laboratories, National Tuberculosis Reference Laboratory, Ministry of Health, Muscat, Sultanate of Oman

**Keywords:** Isoniazid, MDR TB, Gold nanoparticles, *Mycobacterium tuberculosis*

## Abstract

**Background:**

The increased transmission of multidrug-resistant (MDR) tuberculosis (TB) poses a challenge to tuberculosis prevention and control in Sri Lanka. Isoniazid (INH) is a key element of the first line anti tuberculosis treatment regimen. Resistance to INH may lead to development of MDR TB. Therefore, early detection of INH resistance is important to curb spread of resistance. Due to the limited availability of rapid molecular methods for detection of drug resistance in Sri Lanka, this study was aimed at developing a simple and rapid gold nanoparticle (AuNP) based lateral flow strip for the simultaneous detection of the most common INH resistance mutation (*katG* S315 T, 78.6%) and *Mycobacterium tuberculosis* (MTb).

**Methods:**

Lateral flow strip was designed on an inert plastic backing layer containing a sample pad, nitrocellulose membrane and an absorption pad. Biotin labeled 4 capture probes which separately conjugated with streptavidin were immobilized on the nitrocellulose. The test sample was prepared by multiplex PCR using primers to amplify codon 315 region of the *katG* gene and MTb specific *IS6110* region. The two detection probes complementary to the 5′ end of each amplified fragment was conjugated with gold nanoparticles (20 nm) and coupled with the above amplified PCR products were applied on the sample pad. The hybridization of the amplified target regions to the respective capture probes takes place when the sample moves towards the absorption pad. Positive hybridization is indicated by red colour lines.

**Results:**

The three immobilized capture probes on the strip (for the detection of TB, *katG* wild type and mutation) were 100 and 96.6% specific and 100 and 92.1% sensitive respectively.

**Conclusion:**

The AuNP based lateral flow assay was capable of differentiating the specific mutation and the wild type along with MTb identification within 3 h.

## Background

Tuberculosis (TB) is an infectious disease caused by the bacillus *Mycobacterium tuberculosis* (MTb) which remains a major global health problem, responsible for ill health and deaths among millions of people each year. The transmission of multidrug-resistant (MDR) TB poses a challenge to global tuberculosis prevention and control. MDR-TB, defined as resistance to at least rifampicin (RIF) and isoniazid (INH), becomes a crucial factor for the control of the disease, since patients harboring MDR strains of MTb need to be entered into alternative management schedules involving second-line drugs [1–4]. MDR-TB greatly complicates patient management especially within resource-poor national TB programs, by reducing effectiveness of the treatment regime while increasing the cost.

The TB profile of Sri Lanka on World Health Organization (WHO) reports, that in 2016 MDR rate in Sri Lanka was 0.54% among newly diagnosed cases and 3.1% among retreatment cases, with 23 laboratory confirmed MDR TB cases and an estimated TB incidence remaining stable at 65 new cases per 100 000 population [5]. Irrespective of this incidence rate, early diagnosis of MDR TB is essential to control the spread of the disease to reach the goals of the End TB strategy [2].

The phenotypic methods based on conventional cultures that detect growth of MTb in the presence of a particular antibiotic are time consuming. Thus, modern molecular detection methods have been developed for the rapid identification of INH and RIF resistance as well as MTb. An allele-specific PCR targeting *katG* 315 gene and an *inhA *C-15 T mutation in the regulatory region of the *mabA-inhA* operon to detect INH-resistant MTb strains and to identify the *Mycobacterium tuberculosis* Complex (MTC) in the same PCR tube [6] is a successful method reported recently. Detection of MDR using lateral flow nucleic acid biosensors focusing *katG*315 and rpoB531 common mutations [7] and detection of RIF resistance using magnetic nanobead based read out [8] are two other recently reported molecular methods based on padlock probes for determination of drug resistance. An assay based on AuNP to differentiate MTb from MTC using clinical specimens was developed by Soo and co-workers in 2009 [9].

The two line-probe assays for TB, namely the INNO-LiPA Rif.TB assay (Innogenetics NV, Ghent, Belgium) [10–14] and the GenoType MTBDR plus (Hain Lifescience GmbH, Nehren, Germany) [14, 15] have been custom-made to detect mutations causing RIF and INH drug resistance with satisfactory sensitivity and specificity [16, 17]. But it is known that the sensitivity of these tests is lower for INH compared to RIF [18]. In recent times an automated cartridge-based technology (GeneXpert MTB/RIF, Cepheid Inc.) has been introduced for simultaneous detection of MTb and RIF resistance directly from sputum as well as extra pulmonary samples [19, 20]. One obvious disadvantage is the inability of this system to detect INH resistance.

However, the availability of these molecular detection methods is very limited in Sri Lanka as the commercial systems are costly in terms of infrastructure and equipment. Therefore, this study was aimed at developing a simple, convenient and rapid AuNP based lateral flow strip for the simultaneous detection of most common INH resistance mutation (*katG* S315 T) and MTb.

## Methods

### Sample collection

Acid Fast Bacilli (AFB) positive sputum specimens (*n* = 541) were collected from the Central Chest Clinic, Colombo from 2013 to 2016. The sputum specimens were processed and cultured on Lowenstein Jensen medium to isolate MTb strains. A total of 450 cultures were confirmed as MTb using phenotypical characters (growth rate, color and colony morphology) and PCR amplification of *IS6110* insertion element of the MTb genome. Fifteen (15) INH resistant isolates were identified among the 450 isolates by the agar proportion method. In addition, 41 INH resistant MTb strains isolated from sputum samples received from chest clinics were directly collected from National Tuberculosis Reference Laboratory (NTRL), Welisara, Sri Lanka.

### DNA extraction and multiplex PCR amplification

Genomic DNA of INH resistant MTb isolates was extracted using phenol-chloroform method [21]. The extracted DNA was quantified by agarose gel electrophoresis (1.5%) using lambda DNA (539 ng/ μl, Promega).

Multiplex PCR was performed using two specific primer sets to amplify a 217 bp fragment of *katG* gene flanking the codon 315 and a 249 bp fragment of *IS6110* insertion region specific to MTC for identification of INH resistance and MTb [22] simultaneously (Table 1). MTC consists of seven *Mycobacterium* species including MTb, *M. africanum, M. bovis, M. microti, M. canetti, M. caprae* and *M. pinnipedii*.

**Table 1:**
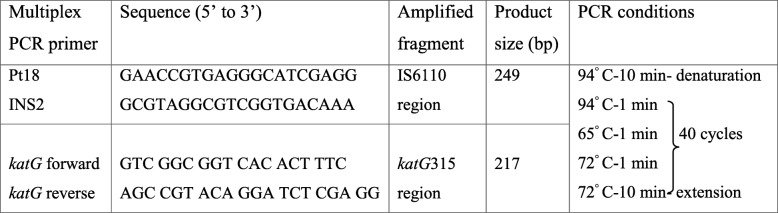
Primers and reaction conditions used for the multiplex PCR amplification of TB specific region and the *katG *315 region

The H37Rv reference MTb strain and a known INH resistant MTb strain confirmed by the NTRL, Welisara, Sri Lanka were used as quality control strains.

### Designing of probes

The AuNP based lateral flow assay requires two types of probes; detection probes conjugated to AuNP and biotin labeled capture probes. In this study two detection probes and four capture probes were designed.

***Detection probe katG (DP katG)*:** Oligonucleotide sequence which is complementary to the 5′ end of the amplified DNA fragment (target) of *katG* gene.

***Detection probe for TB (DP TBD)*:** Oligonucleotide sequence which is complementary to the 5′ end of the amplified DNA fragment of *IS6110* insertion region.

***Capture probe for wild type and the mutated strains detection (CP WT and CP ACC):*** Oligonucleotide sequence which is complementary to the 3′ end of the target amplified DNA fragment of *katG* gene. These two probes were designed focusing the codon 315 position where the most common point mutation occurs.

***Capture probe for TB detection (CP TBD):*** Oligonucleotide sequence which is complementary to the 3′ end of the target amplified DNA fragment of MTb specific region.

***Capture probe for control (CP Control):*** Oligonucleotide sequence complementary to the DP TBD detection probe. Hybridization of DP TBD to the CP control will verify the assay performance.

These probes were designed using IDT Oligo Analyzer 3.1 tool to contain a poly A tag and thiol or biotin modifications as described in previous procedures [9, 23, 24] (Table 2). The poly A tag was introduced to the 5′ end of the detection probes and to the 3′ end of each of the capture probes. Detection probes were thiol-modified and conjugated with AuNPs under optimized conditions. Thiol modification facilitated the stronger binding of AuNP to the detection probe via the formation of strong Au-S bonds [7, 8]. Capture probes were labeled with biotin for the easy conjugation with streptavidin.

**Table 2:** Oligonucleotides used in the development of the assay

Probe name	Description	Sequence (5′ to 3′) with modification
CP WT	Wild type probe (strip oligo)	GATCACCAGCGGCATCGAGAAAAAAAAAA -Biotin
CP ACC	Mutation probe (strip oligo)	GATCACCACCGGCATCGAGAAAAAAAAAA -Biotin
CP TBD	TB detection probe (strip oligo)	GGC TGT GGG TAG CAG ACC AAAAAAAAAA -Biotin
CP Control	Control probe (strip oligo)	CCGTTCGACGGT GCA TCT G AAAAAAAAAA -Biotin
DP TBD	TB detection probe (with AuNP)	HS-AAAAAAAAAACAGATGCACCGTCGAACGG
DP *katG*	*katG* detection probe (with AuNP)	HS-AAAAAAAAAAGCTGGAGCA GATGGGCTT

### Synthesis, purification, and characterization of gold nanoparticles

**Fig. 1:**
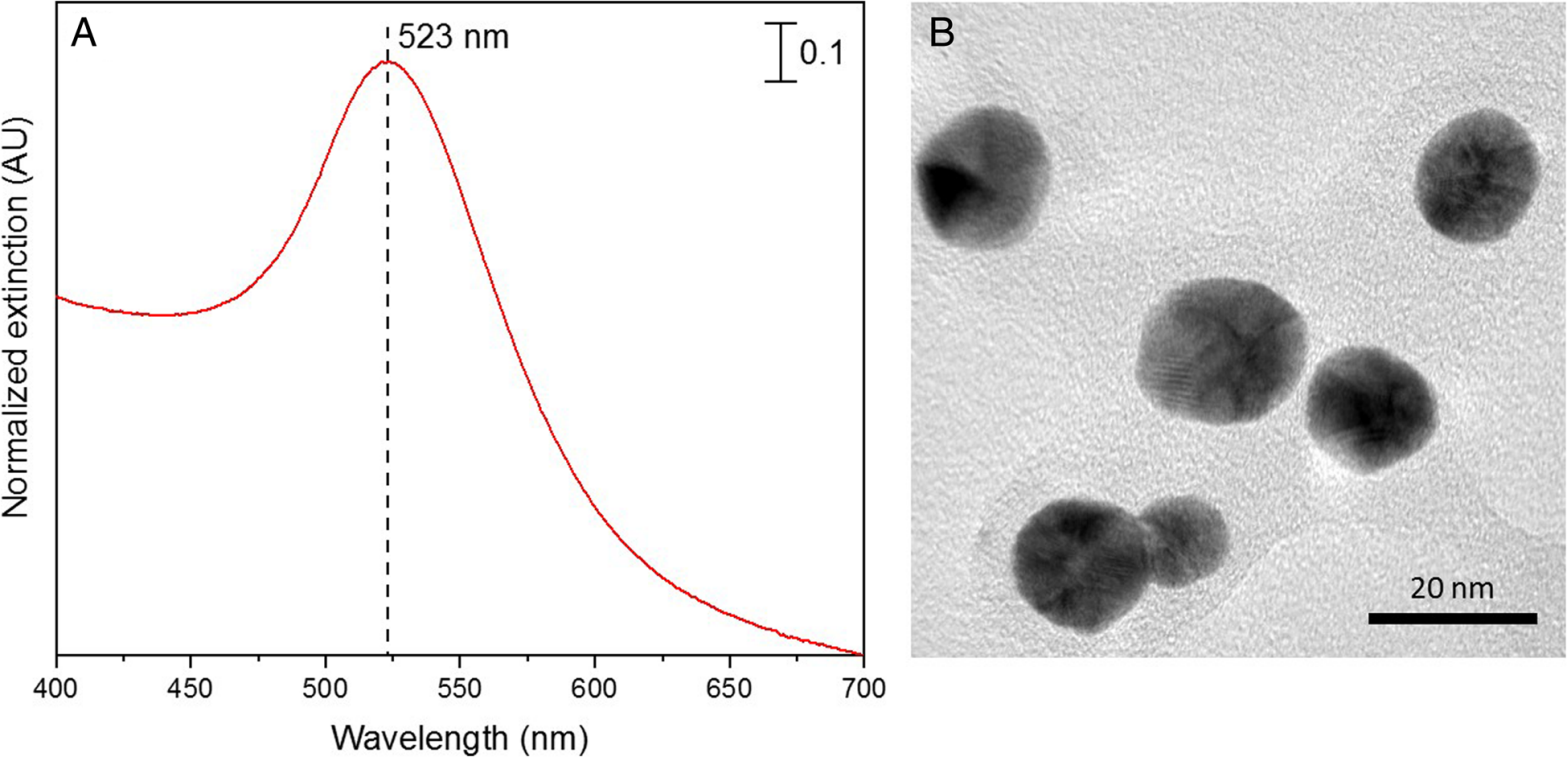
(**a**) Localized Surface Plasmon Resonance (LSPR) spectrum and (**b**) a representative HRTEM image of gold nanoparticles

AuNPs were prepared according to the previously described citrate method [23, 25]. First, all glassware were thoroughly cleaned with aqua regia (three parts conc. HCl, one part conc. HNO_3_), rinsed with copious amounts of water followed by nanopure water, and oven-dried prior to use. In a 500 mL round-bottom flask, 200 mL of 1 mM HAuCl_4_ prepared in nanopure water was brought to a boil with vigorous stirring under continuous condensation. To this solution, 4 mL of 39 mM trisodium citrate was added. The solution turned deep blue within 20 s, and finally the color changed to wine-red few minutes later indicating the formation of AuNPs. Boiling was continued for an additional 10 min to ensure complete reaction and the colloid solution was then stirred for further 15 min while allowing it to cool to room temperature. The resulting AuNP suspension was aliquoted into 2 mL volumes and centrifuged at 6000 rpm for 1 h to remove any unreacted material from the supernatant. The separated AuNP pellet was then diluted using a 0.5 mM citrate storage buffer back to the initial volume (2 mL) and the AuNPs were re-dispersed in solution followed by the centrifugation at 6000 rpm for another 1 h. This step was repeated for 3 times to ensure the complete removal of unreacted materials in the as-synthesized media and hence increased the stability of AuNPs. Localized Surface Plasmon Resonance spectrum (LSPR) of purified Au-NPs indicated a λ_max_ of 523 nm [7] as shown in Fig. 1 (A). According to the approach previously developed by Haiss and co-workers [26], this LSPR behavior evidenced the presence of gold nanoparticles of ~ 20 nm average diameter, at a ~ 2.5 nM particle concentration. Further, the narrow spectral bandwidth and the absence of any secondary spectral features in the LSPR spectrum of the synthesized AuNP, subsequent to purification, indicated good homogeneity in particle size and appreciable stability under the experimental conditions. Additionally, high resolution transmission electron microscopic (HRTEM – JEOL JEM 2100) images of the prepared AuNPs were obtained to reconfirm the size of the AuNPs as shown in the representative HRTEM micrograph image given in Fig. 1 (B). The HRTEM indicated the presence of spherical and monodisperse gold nanoparticles of ~ 20 nm diameter, reconfirming the inference made via the LSPR spectrum. Importantly, these particles were very stable in solution and no changes could be observed in the LSPR spectra even after several weeks post-synthesis. Hence, the AuNPs were stored at 4 °C until they were utilized in the conjugation procedure.

### Conjugation of AuNPs to detection probes

The preparation of DNA conjugated AuNPs was modified from the procedure previously described [24]. First, 20 μL of 100 μM thiol–modified oligonucleotides were gently added to 500 μL of colloidal gold. The solution was incubated at 4 °C in the dark conditions for 24 h. Then the solution was added to 10 mM phosphate buffer (pH = 7). A 30 μL volume of 1 M NaCl solution was added drop wise to the solution and centrifuged at 12000 rpm for 20 min. The resulting conjugated AuNPs were blocked in 500 μL of 5% Bovine Serum Albumin (BSA) solution for 30 min at 25 °C followed by 12000 rpm centrifugation for 15 min. The final colloid sample was re-dispersed in 500 μL of sample eluent buffer (10% sucrose, 20 mM Na_3_PO_4,_ 5% BSA, 0.25% Tween 20) [23]. The addition of BSA, tween 20 and sucrose facilitates the stability of AuNPs. Conformation of the conjugation of AuNPs to detection probes was done using spectrophotometric analysis [7, 8]. A shift of LSPR λ_max_ from 523 nm to 526 nm showed that the prepared AuNPs were conjugated with the oligonucleotides.

### Conjugation of streptavidin to capture probes

Conjugation of streptavidin to biotin labeled capture probes was done using the previously described procedure [23] with slight modifications. Briefly, 1 mg of streptavidin was dissolved in 900 μL of 1x PBS. A 220 μL volume of this solution was mixed with 20 μL of 1 mM capture probe in a microcentrifuge tube and incubated 1 h at 25 °C and 1 h at 37 °C. Excess probes were removed by 30,000 cut off centrifugal filter (Millipore) by centrifugation at 6000 rpm. Resulted conjugates were washed with 300 μL of 1x PBS two times in the same centrifugal filter. Remaining solution of the filter was eluted into a new microcentrifuge tube and stored at 4 °C.

### Lateral flow strip preparation

**Fig. 2:**
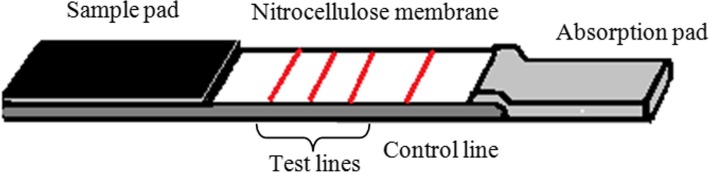
Schematic illustration of the lateral flow strip

Lateral flow strip was designed to contain a sample pad, test zone and an absorption pad mounted on an inert plastic backing layer. The test zone includes separate test lines for mutation probe, wild type probe*,* MTb identification probe and the control probe (Fig. 2). The sample pad was prepared by saturating a cellulose pad with 100 μL of sample pad saturation buffer (0.25% Triton X100, 0.05 M Tris HCl, 0.15 M NaCl, pH = 8) [23]. It was dried and stored in a dessicator. Absorption pad was also a cellulose pad which was used to facilitate the flow of solutions throughout the strip via capillary action. Test zone on the nitrocellulose membrane was used to immobilise the streptavidine conjugated biotin labelled capture probes as test lines and a control line. A 0.6 μL volume of each streptavidin conjugated capture probe was spotted as a line using a micro pipette and the membrane was incubated 16 h at 37 °C. The nitrocellulose membrane was blocked with 50 μL of membrane blocking buffer (1% sucrose, 1% BSA, 2.5% Tween 20, 0.1 M NaH_2_PO_4_) and incubated at 37 °C for 30 min. The sample pad, nitrocellulose membrane and the absorption pad were assembled on a plastic backing layer to form the lateral flow strip (Fig. 2).

### Assay procedure

A 5 μL volume of multiplex PCR amplified product was denatured at 99 °C for 8 min and immediately cooled in ice for 5 min. Then, 10 μL of each of the TB detection and *katG* detection probes were mixed with the PCR amplified, denatured targets (*katG* gene fragment and *IS6110* insertion region fragment) and hybridized at 42 °C for 15 min in a microcentrifuge tube. The resulting hybridized mixture was introduced on to the sample pad followed by the addition of 40 μL of running buffer (1% BSA, 0.2% tween20, 0.01% SDS in 6x SSC) [23]. The capture probes on the strip were then allowed to hybridize to the above mixture at 48 °C for 15 min and were washed with 4x SSC buffer.

### Limit of detection

Concentration of the extracted target genomic DNA was determined using Quantus fluorometer (Promega). Twofold serial dilution was done for the target DNA starting with 32 pg/ μL (H37Rv strain) and 50 pg/ μL (INH resistant reference strain). Thereafter, multiplex PCR was carried out for all dilutions (five dilutions of each strain). The resulted multiplex PCR products were denatured and subjected to the above assay procedure. The strips were prepared individually for each capture probe (TBD, WT and ACC) along with the control probe. The multiplex PCR products of H37Rv strain was used to determine the limit of detection (LOD) of TBD and WT capture probes while PCR product of the reference mutant strain was used to determine the LOD of ACC capture probe.

### Validation of the assay

First, specificity of the assay was performed using DNA of five different species of non-tuberculous mycobacteria (NTM) (*M. abscessus, M. fortuitum, M. avium*, *M. smegmatis*, *M. kansasii*). These five isolates are used as the reference NTM standards in the laboratory and have been confirmed as NTM by biochemical testing and DNA sequencing.

Then, the assay was evaluated as a molecular method for simultaneous identification of both TB and INH resistance. The mutant probe was validated using 38 INH resistant MTb isolates which were prior confirmed using agar proportion method followed by DNA sequencing. Wild type probe was validated using 20 INH susceptible isolates confirmed by agar proportion method. The validation of the TB detection probe was carried out using all 58 MTb isolates. They were confirmed as MTb by PCR amplification of the 240 bp fragment of the *IS6110* element.

The specificity of the TB detection probe was determined using 10 NTM isolates (other than the 5 isolates mentioned above). These NTM isolates produced negative results for *IS6110* fragment PCR amplification confirming absence of MTb. Specificity of the mutant probe and wild type probe was determined using the above 20 INH susceptible isolates and 38 INH resistant isolates respectively [14]. The H37Rv reference MTb strain as well as the confirmed INH resistant isolate by NTRL was used as the controls.

Sensitivity and the specificity of each of the three probes were statistically calculated using SPSS 18 software.

## Results and discussion

**Fig. 3:**
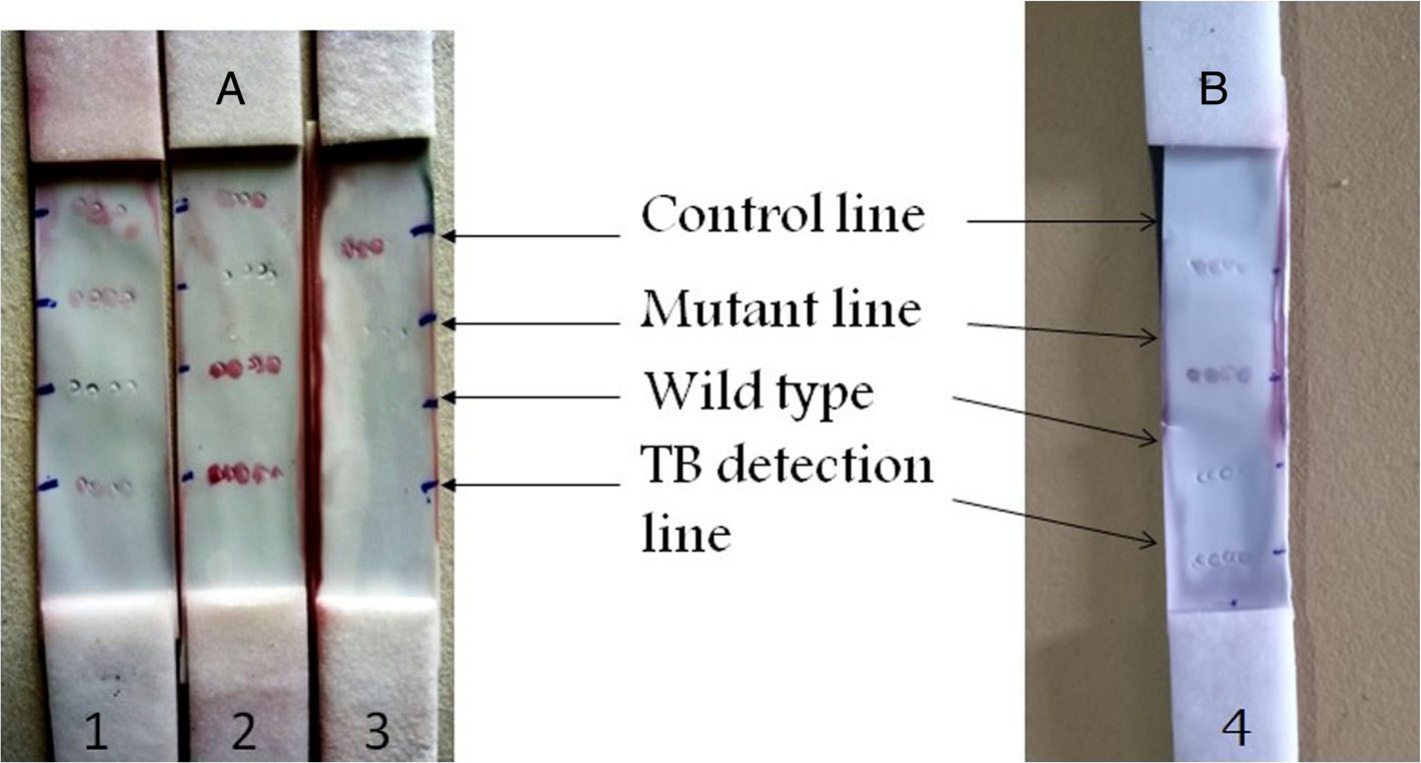
Lateral flow assay strip run with DNA of reference INH resistant isolate (A1), wild type H37Rv (A2), NTM isolate (A3) and INH resistant test isolate (B4)

The lateral flow assay done using the reference and test isolates are shown in Fig. 3 (3A and 3B respectively). The control line appeared within ~ 20 min and the test lines became visible in ~ 35 min. Thus the total assay time is approximately 35 min.

**Fig. 4:**
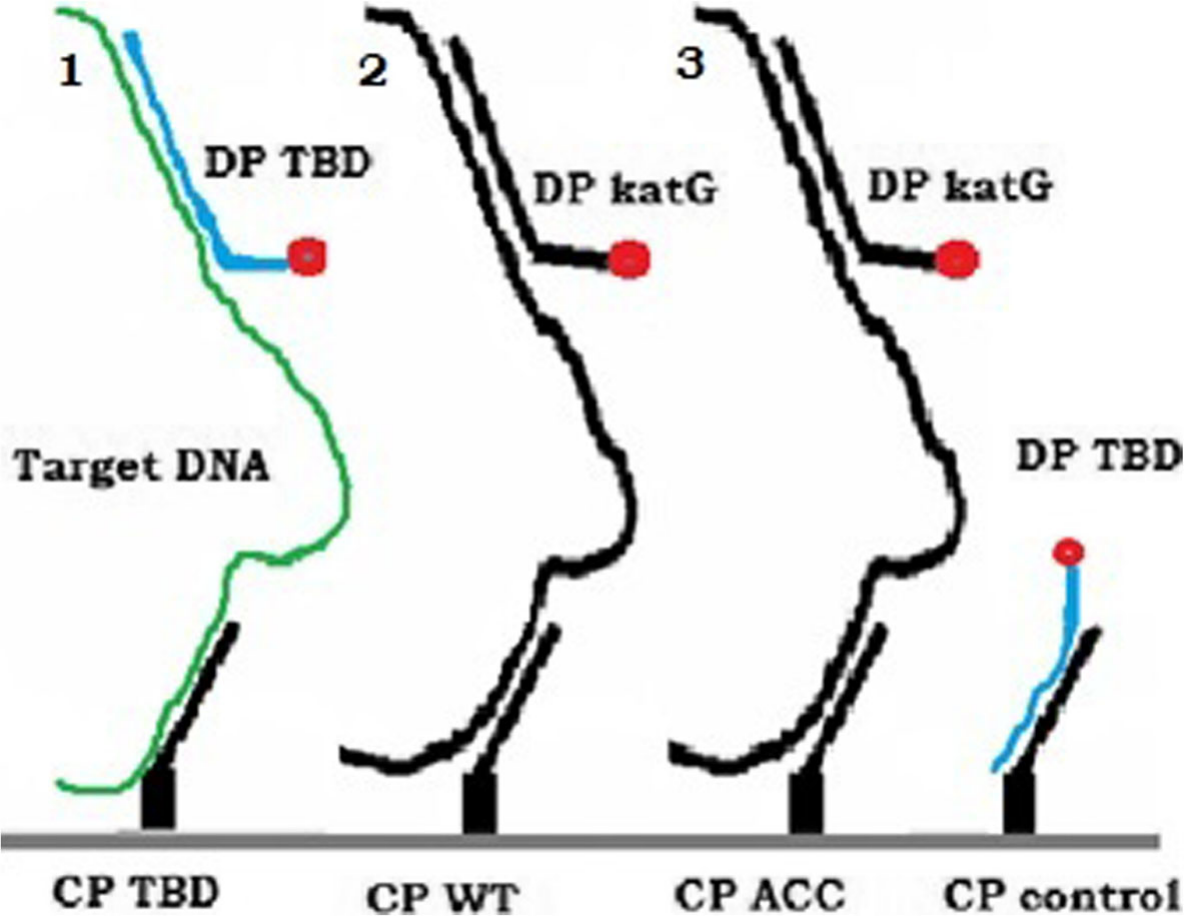
Schematic illustration of the lateral flow assay (DP- detection probe, CP- capture probe, TBD- Tb detection, katG – *katG* detection, WT-wild type *katG,* ACC- mutant *katG*,1- *IS 6110* PCR fragment, 2 & 3- *katG* gene PCR fragments)

In the assay procedure, both detection probes (DP *katG* and DP TBD conjugated to AuNPs) were hybridized to 5′ end of the each of the two mobile target DNA fragments in a microcentrifuge tube. The 3′ end of the mobile target DNA (carrying the detection probes) would then hybridize to the immobilized capture probes on the nitrocellulose membrane resulting in a red color at the relevant test line due to the accumulation of AuNPs conjugated to detection probes (Fig. 4). The excess TBD detection probe that flows through the nitrocellulose membrane will then hybridize with the complementary control capture probe. A positive signal at the control line confirms the correct direction of the flow.

The Table 3 shows the LOD of each of the capture probes individually using reference H37Rv strain and the INH resistant mutant strain. The PCR product of the lowest DNA concentration that produced an intense red colour band was recorded as the LOD of each of the probe. However, LOD for the overall assay was taken as 6.25 pg/μL (LOD of CP ACC) since all probes were immobilized on a single strip.

**Table 3:** Limit of detection of each capture probe

DNA of the isolate	CP TBD	CP WT	CP ACC
H37Rv	2 pg/μL	4 pg/μL	–
Reference mutant strain	3.125 pg/μL	–	6.25 pg/μL

When the specificity of the assay performed using DNA of reference NTM species (*M. abscessus, M. fortuitum, M. avium*, *M. smegmatis*, *M. kansasii*), all five NTM species showed a positive band only for the control line confirming 100% specificity.

The details of the evaluation of the performance of assay as a molecular method for simultaneous identification of both TB and INH resistance is shown in Table 4.

**Table 4:** Validation of the assay strip using INH resistant, susceptible and NTM isolates

Isolates	Mutant probe (CP ACC)		WT probe (CP WT)		TB detection probe (CP TBD)	
	*P*	*N*	*P*	*N*	*P*	*N*
INH resistant (*n* = 38)	35	3	0	38	36	2
INH susceptible (*n* = 20)	0	20	20	0	20	0
NTM (*n* = 10)	0	10	0	10	0	10
False positive	–		–		–	
False negative	3 (4.4%)		–		2 (2.9%)	

*P* Positive, *N* Negative

With the lateral flow assay out of the 68 isolates, 65 isolates revealed expected results providing 96.6% compatibility. Three isolates gave false negative results for the detection by the mutant probe. Of these three isolates two isolates were also negative for the TB detection probe (Table 4).

Sensitivity and the specificity of the assay for each of the three capture probes are shown in Table 5.

**Table 5:** Sensitivity and specificity of the three capture probes

Probe	Specificity (%)	Sensitivity (%)	Positive predictive value (%)	Negative predictive value (%)
Mutant probe (CP ACC)	100.0 (C.I. 84.6–100.0)	92.1 (C.I. 84.0–92.1)	100.0 (C.I. 90–100)	87.0 (C.I. 66.4–97.2)
Wild type probe (CP WT)	100.0 (C.I. 93.1–100.0)	100.0 (C.I. 86.8–100.0)	100.0 (C.I. 86.8–100)	100.0 (C.I. 93.1–100.0)
TB detection probe (CP TBD)	100.0 (C.I. 71.9–100.0)	96.6 (C.I. 91.7–96.6)	100.0 (C.I. 93.1–100)	83.3 (C.I. 51.6–98.0)

In the optimization process of the assay, the hybridization temperature of each probe was initially determined. Thereafter, the hybridization temperature for the strip was optimized to a single temperature since all probes were immobilized on the same strip. Flushing 4x SSC buffer over the strip as a washing process facilitated the specific binding of each of the probe. A clear background was obtained when freshly prepared membrane blocking buffer was used after immobilization of the capture probes on to the nitrocellulose membrane. The components in the sample pad saturation buffer would facilitate the flow of DNA conjugates towards the adsorption pad by reducing entrapment of target DNA conjugates in the sample pad.

Most lateral flow strips contain a conjugate pad saturated with the AuNPs conjugated detection probes, which is placed in between the sample pad and the nitrocellulose membrane [23, 27, 28]. But, in the current method, the hybridization of target DNA sample with the AuNPs conjugated detection probes was done in a separate micro-centrifuge tube to achieve maximum hybridization between the target and the AuNPs conjugate. Since this hybridized solution was applied on to the sample pad of the strip, it facilitated the hybridization with immobilized capture probes enhancing the color intensity of the bands. In the current assay, a cellulose pad was used as the sample pad instead of a glass fiber pad to reduce the cost.

The maximum storage time of the developed strip was 2 weeks in a desiccator at room temperature (25 °C), but use of a clamshell laminator to assemble the strip on the plastic backing layer will be a more efficient way to increase the expiry date. Additionally, it will shorten the time required for manual preparation of assay strips. The manual dispensing of the capture probes as spots in a line using a micropipette on the nitro cellulose membrane was time consuming and laborious. Use of air jet dispensers will be a more efficient method and it will minimize the amount of capture probes required per strip.

Even though, the strip was developed manually using minimum resources which were available in the laboratory, it was adequate to discriminate between mutant and wild types efficiently. A few INH resistant isolates (*n* = 4), gave a very weak positive wild type line along with the intense mutant line. This was acceptable because some of the pure MTb cultures could contain a mixture of both mutated and wild type strains [29].

Although the TB detection conjugate probe was validated using MTb isolates, any one of the seven Mycobacterium species of MTC can be detected by the strip since the TB detection capture probe was designed based on gene sequence in *IS6110* insertion element common to MTC.

This assay was developed for the detection of the most common *katG*315 (AGC to ACC) mutation (78.6%) [30] responsible for INH resistance in Sri Lanka. But the strip can be further developed to detect other less common INH resistant mutations (AGC to AAC/AGA) in Sri Lanka by immobilizing the relevant mutation specific capture probes on to the strip, so that custom made strips can be prepared depending on the frequency and types of mutations in a particular geographical setting. Furthermore, the strip can be developed to detect MDR TB by introducing a specific probe to detect RIF resistance using most common *rpoB*526 mutation (CAC to TAC) reported in Sri Lanka [31].

## Conclusion

This AuNP based lateral flow assay is appropriate for INH resistance screening in low resource settings as it reliably differentiates the resistant mutation from the wild type simultaneously with MTb identification within 3 h time beside its convenient use.

## Data Availability

The datasets used and/or analyzed during the current study are available from the corresponding author on reasonable request.

## Abbreviations

AuNP: gold nanoparticle
BSA: Bovine Serum Albumin
CP ACC: Capture probe for mutated *katG315*
CP Control: Capture probe for control
CP TBD: Capture probe for TB detection
CP WT: Capture probe for wild type *katG315*
DP *katG*: Detection probe *katG*
DP TBD: Detection probe for TB
HRTEM: High resolution transmission electron microscope
INH: Isoniazid
LOD: Limit of detection
LSPR: Localized Surface Plasmon Resonance spectrum
MDR: multidrug-resistant
MTb: *Mycobacterium tuberculosis*
MTC: *Mycobacterium tuberculosis* complex
NTM: Non-tuberculous mycobacteria
NTRL: National Tuberculosis Reference Laboratory
PCR: Polymerase chain reaction
RIF: Rifampicin
TB: tuberculosis
